# How dieting might make some fatter: modeling weight cycling toward obesity from a perspective of body composition autoregulation

**DOI:** 10.1038/s41366-020-0547-1

**Published:** 2020-02-25

**Authors:** Philippe Jacquet, Yves Schutz, Jean-Pierre Montani, Abdul Dulloo

**Affiliations:** 1 Department of Endocrinology, Metabolism & Cardiovascular system, Faculty of Science & Medicine, University of Fribourg, Fribourg, Switzerland; 2 Scientific Computing and Research Support Unit, Computer Center, University of Lausanne, Lausanne, Switzerland

**Keywords:** Fat metabolism, Medical research

## Abstract

The notion that dieting makes some people fatter has in the past decade gained considerable interest from both epidemiological predictions and biological plausibility. Several large-scale prospective studies have suggested that dieting to lose weight is associated with future weight gain and obesity, with such predictions being stronger and more consistent among dieters who are in the normal range of body weight rather than in those with obesity. Furthermore, the biological plausibility that dieting predisposes people who are lean (rather than those with overweight or obesity) to regain more body fat than what had been lost (referred to as fat overshooting) has recently gained support from a re-analysis of data on body composition during weight loss and subsequent weight recovery from the classic longitudinal Minnesota Starvation Experiment. These have revealed an inverse exponential relationship between the amount of fat overshot and initial adiposity, and have suggested that a temporal desynchronization in the recoveries of fat and lean tissues, in turn residing in differences in lean-fat partitioning during weight loss vs. during weight recovery (with fat recovery faster than lean tissue recovery) is a cardinal feature of fat overshooting. Within a conceptual framework that integrates the relationship between post-dieting fat overshooting with initial adiposity, the extent of weight loss and the differential lean-fat partitioning during weight loss vs. weight recovery, we describe here a mathematical model of weight cycling to predict the excess fat that could be gained through repeated dieting and multiple weight cycles from a standpoint of body composition autoregulation.

## Background

In parallel to the increasing prevalence of overweight and obesity worldwide, the prevalence of dieting is also rising, and current estimates indicate that 40% of adults have tried to lose weight at some point during the last 5 years [1]. With studies of the long-term outcomes showing that at least one-third of dieters regain more weight than they lost [2], together with prospective studies indicating that dieting—whether in adults [3–11], adolescents [12–16] or children [17–19]—predicts future weight gain and obesity, there is concern as to whether dieting may paradoxically be promoting exactly the opposite of what it is intended to achieve [20–22].

Indeed, the notion that dieting to lose weight is counterproductive for weight management in that people may regain more fat than they lose through each cycle of weight loss/regain was embodied in the title of a book published in 1983: ‘*Dieting makes you fat*’ [23]. This notion remains controversial and the subject of frequent debates among scientists [24–31] despite the conclusion of a US National Task Force on the Prevention and Treatment of Obesity [32] that to quote: ‘*the available evidence is not sufficiently compelling to override the potential benefits of moderate weight loss in significantly obese patients*’.

While this conclusion made 25 years ago may still be valid today, its specification pertaining to patients with obesity was perhaps a premonition for the subsequent findings from several prospective studies suggesting that it was dieting in people of normal body weight, rather than in those with overweight or obesity, that was most strongly and consistently associated with future weight gain [33, 34] and risks for cardiometabolic diseases [34]. In particular, in a 6–15-year follow-up study in young and middle-aged population groups, the risk of major weight gain exceeding 10 kg was found to be higher (by twofold) in dieters than in non-dieters among those initially of normal body weight but not among those initially with overweight or obesity [6]. In another large population-based cohort with a 10-year follow-up from adolescence to young adulthood [16], a dose-dependent association was found between the frequency of intentional weight loss episodes of more than 5 kg and the gain in body mass index (BMI) over the 10-year follow-up, as well as with the risk of overweight at 25 years of age. Furthermore, those in the lowest BMI category at baseline gained more weight than those in the intermediate or high baseline BMI category [16]. More recently, in a study based on a representative adult population sample (Finnish Health 2000 Survey) and on its follow-up examination 11 years later in 2,785 adults aged 30–69, the increases in BMI and waist circumference were found to be greater in dieters than in non-dieters, but notably greatest in dieters who reported that they had lost weight or experienced weight fluctuation during the previous year, and in dieters with initially normal weight [11].

Taken together, these studies reinforce the contention that dieting to lose weight and weight cycling most strongly predict future weight gain in those who are lean than in those who are with overweight/obesity. By extension, they reinforce the plausibility that at least in the people without obesity, each cycle of weight loss/regain is accompanied by a greater gain of body fat than is lost. Such a phenomenon referred to as weight (or fat) overshooting is directly supported by the classic longitudinal study of semistarvation and refeeding—the Minnesota Experiment [35]—conducted in young men in the normal range of BMI. During their nutritional rehabilitation after losing 25–29% of their body weight over 24 weeks of semistarvation, they showed fat overshooting of 4 kg on average but ranging between 0 and 9 kg, with those showing higher fat overshooting being the leanest, as evidenced from an inverse exponential relationship between the kg of fat overshot and initial (pre-starvation) body fat% [36]. Thus, a high dependency of post-dieting fat overshooting upon the initial adiposity is a central tenet in explaining the findings of prospective studies showing a more consistent association between dieting to lose weight and increased risks for major weight gain in individuals initially of normal-weight than in people initially with overweight or obesity [6, 11, 16].

Against this background, we describe here the development and application of a mathematical model to predict the amount of fat overshoot through multiple weight cycles in pathways from leanness to fatness—albeit from a standpoint of body composition autoregulation.

## Development of the model

### Basic concepts

Several mathematical models have been developed to study the regulation of body weight and body composition in which the initial body composition is a simple function that determined the fraction of energy imbalance partitioned toward deposition or mobilization of body protein vs. fat [37–42]. The model presented here, however, rests upon the notion that the initial body composition could also be a factor in the mechanisms by which weight cycling might predispose people to increased fatness. The basic concepts here underlying this modeling of weight cycling from leanness to fatness rests upon several findings from our previous re-analysis of data from the Minnesota Experiment on changes in body composition, energy intake and basal metabolic rate in the 32 men who completed the 24 weeks of semistarvation and 12 weeks of controlled refeeding, as well as in the 12 subjects who also completed the subsequent 8 weeks of refeeding with *ad libitum* access to food. These are summarized below:

(i) During weight loss in response to semistarvation, an intrinsic lean-fat partitioning characteristic of the individual (Pss) dictates the relative proportion of body energy derived from fat-free mass (FFM), and that this characteristic, which is conserved during refeeding, is a function of the initial body fat% [43, 44]. This is consistent with the theoretical equation developed earlier by Forbes [41] that quantified the non-linear relationship between the fat-free proportion of modest weight changes as a function of the initial body fat, and later extended by Hall [42] to account for the magnitude of body weight changes.
(ii) An adaptive suppression of thermogenesis, which operates to conserve energy during weight loss, persists as a function of fat depletion during weight recovery and serves to accelerate specifically the recovery of fat mass but not that of FFM [43, 45].
(iii) The hyperphagia during ad libitum refeeding is driven not only by the degree of fat depletion, but also by the degree of FFM depletion [46].
(iv) The operation of these above-mentioned control systems during refeeding is that body fat recovery reaches completion (to baseline pre-starvation levels) before full recovery of FFM, and that hyperphagia (which is partly driven by FFM depletion) persists until complete FFM recovery, with concomitant accumulation of excess fat and hence fat overshoot [46]. In other words, because of the temporal desynchronization in the complete recovery of fat and FFM, fat overshoot is a prerequisite that enables the recovery of FFM driven by hyperphagia to be completed – a process that is referred to as collateral fattening [47, 48]. In turn, it can be hypothesized that the temporal desynchronization between completion in fat and FFM recoveries reside in differences in lean-fat partitioning during weight loss vs. during weight recovery.

### Mathematical modeling of fat overshoot

**Fig. 1:**
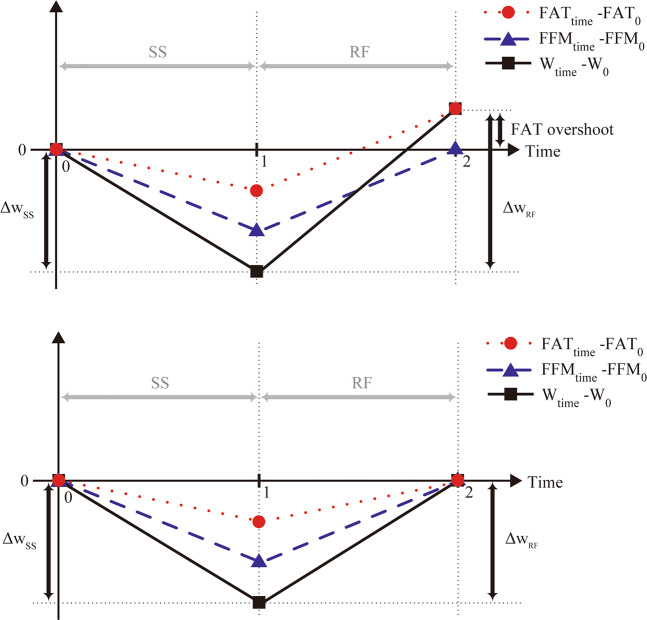
Changes in body weight (W), body fat (FAT) and fat-free mass (FFM) in response to semistarvation (SS) and subsequent refeeding (RF); time 0, 1 and 2 represent ‘prior to semistarvation’, at the ‘end of the semistarvation’ period, and at the end of the refeeding period when body weight has been completely recovered, respectively. The dynamics of body composition recovery are depicted with FAT and FFM recoveries being desynchronized and resulting in fat overshooting (*upper panel*) or synchronized so as to reach 100% recoveries at the same time without fat overshooting (*lower panel*). All values are expressed as a difference from the corresponding values during the control (time 0) period. More precisely, the values are defined as $FAT_{{\mathrm{time}}} - FAT_0$ for FAT (red circle, dotted line), $FFM_{{\mathrm{time}}} - FFM_0$ for FFM (blue triangle, dashed line) and $W_{{\mathrm{time}}} - W_0$ for W (black square, solid line).

On the basis of the above basic concepts derived from the re-analysis of data from the Minnesota Experiment, we start the modeling of fat overshooting by depicting in Fig. 1 the simulation of changes in body weight and body composition of a subject of normal body weight who goes through the two successive phases of a weight cycle. In the first phase (time 0–1), the subject loses weight through semistarvation (SS), and in the second phase (time 1–2), the subject regains weight through refeeding (RF) until complete recovery of FFM, namely until $FFM_2 = FFM_0$. It is to be noted that (i) it is assumed that at time 0, 1 and 2 the body fat (FAT) and fat-free mass (FFM) are known, and (ii) the lines in Fig. 1 are only added to ‘guide the eyes’, and no assumption is made that the weight loss or gain is linear in time. The body weight of the subject across time is then defined as: $W_{{\mathrm{time}}} = FAT_{{\mathrm{time}}} + FFM_{{\mathrm{time}}}$, with time = 0, 1 or 2.

As shown in Fig. 1, the subject has an initial weight $W_0 = FAT_0 + FFM_0$, where *FAT*_0_ and *FFM*_0_ are the subject’s initial FAT and FFM contents, respectively. Between time 0 and 1, the subject loses weight Δ*W*_SS_ > 0 during semistarvation, and reaches at time 1 at the weight given as:

1 $$W_1 = W_0 - \Delta W_{{\mathrm{SS}}} = FAT_1 + FFM_1,$$

where *FAT*_1_ and *FFM*_1_ are his FAT and FFM contents at time 1, respectively.

The weight loss may be written as $\Delta W_{{\mathrm{SS}}} = \Delta FAT_{{\mathrm{SS}}} + \Delta FFM_{{\mathrm{SS}}}$, where Δ*FAT*_SS_ and Δ*FFM*_SS_ are the FAT and FFM, respectively, that are lost during the semistarvation process. These quantities may be computed as $\Delta FAT_{{\mathrm{SS}}} = FAT_0 - FAT_1$ and $\Delta FFM_{{\mathrm{SS}}} = FFM_0 - FFM_1$. Furthermore, the lean-fat partitioning ratio $P_{{\mathrm{SS}}}^{\mathrm{m}}$ during the semistarvation phase is defined as the fraction of the weight loss as FFM, that is $\Delta FFM_{{\mathrm{SS}}} = P_{{\mathrm{SS}}}^{\mathrm{m}} \cdot \Delta W_{{\mathrm{SS}}}$. Thus, $P_{{\mathrm{SS}}}^{\mathrm{m}}$ can be written as:

2 $$P_{{\mathrm{SS}}}^{\mathrm{m}} = \frac{1}{{1 + \frac{{\Delta FAT_{{\mathrm{SS}}}}}{{\Delta FFM_{{\mathrm{SS}}}}}}}.$$

During the refeeding phase (time 1–2) presented in Fig. 1, the subject goes through a weight regain process until his FFM is completely recovered, that is until $FFM_2 = FFM_0$. The weight regained during refeeding can be written as Δ*W*_RF_ > 0, such that $W_2 = W_1 + \Delta W_{{\mathrm{RF}}}$. As in the semistarvation phase, one may write $\Delta W_{{\mathrm{RF}}} = \Delta FAT_{{\mathrm{RF}}} + \Delta FFM_{{\mathrm{RF}}}$, where $\Delta FAT_{{\mathrm{RF}}} = FAT_2 - FAT_1$ and $\Delta FFM_{{\mathrm{RF}}} = FFM_2 - FFM_1$, and introduce the refeeding lean-fat partition ratio $P_{{\mathrm{RF}}}^{\mathrm{m}}$ as the fraction of the weight regained as FFM, that is $\Delta FFM_{{\mathrm{RF}}} = P_{{\mathrm{RF}}}^{\mathrm{m}} \cdot \Delta W_{{\mathrm{RF}}}$. Thus, $P_{{\mathrm{RF}}}^{\mathrm{m}}$ can be written as:

3 $$P_{{\mathrm{R}}F}^{\mathrm{m}} = \frac{1}{{1 + \frac{{\Delta FAT_{{\mathrm{R}}F}}}{{\Delta FFM_{{\mathrm{R}}F}}}}}.$$

Using the equations above, Δ*W*_RF_ can be written as follows:

4 $$\begin{array}{*{20}{c}} {\Delta W_{{\mathrm{RF}}}} = {\frac{{\Delta FFM_{{\mathrm{RF}}}}}{{P_{{\mathrm{RF}}}^{\mathrm{m}}}}} \\ {} = {\frac{{FFM_2 - FFM_1}}{{P_{{\mathrm{RF}}}^{\mathrm{m}}}}} \\ {} = {\frac{{FFM_0 - FFM_1}}{{P_{{\mathrm{RF}}}^{\mathrm{m}}}}} \\ {} = {\frac{{\Delta FFM_{{\mathrm{SS}}}}}{{P_{{\mathrm{RF}}}^{\mathrm{m}}}}} \\ {} = {\frac{{P_{{\mathrm{SS}}}^{\mathrm{m}} \times \Delta W_{{\mathrm{SS}}}}}{{P_{{\mathrm{RF}}}^{\mathrm{m}}}}} \\ {} = {\gamma \times \Delta W_{{\mathrm{SS}}},} \end{array}$$

where

5 $$\gamma = \frac{{P_{{\mathrm{SS}}}^{\mathrm{m}}}}{{P_{{\mathrm{RF}}}^{\mathrm{m}}}}.$$

As it will become evident in later sections, the factor *γ*, which relates the lean-fat partitioning of the subject during weight loss with that during weight regain, plays a fundamental role in FAT overshooting.

The FAT overshoot corresponds to the excess FAT deposited during the refeeding phase as compared with the initial time 0, and can be described as follows:

$$\begin{array}{*{20}{c}} {FAT\,Overshoot} = {FAT_2 - FAT_0} \\ {} = {{W}_2 - {W}_0} \\ {} = {{W}_1 + \Delta {W}_{\mathrm{RF}} - {W}_0} \\ {} = {{W}_0 - \Delta {W}_{{\mathrm{SS}}} + \Delta {W}_{{\mathrm{RF}}} - {W}_0} \\ {} = {\Delta {W}_{{\mathrm{RF}}} - \Delta {W}_{{\mathrm{SS}}}.} \end{array}$$

Thus, using the Eq. (4), we obtain:

6 $$FAT\,Overshoot = \left( {\gamma - 1} \right) \times \Delta W_{{\mathrm{SS}}}.$$

This is a key equation in the modeling process. It is an exact mathematical relation without approximation (i.e., not derived from any data) and the parameter *γ* is a cardinal feature in the process leading to fat overshooting. If *γ* = 1, then the lean-fat partitioning during weight loss is the same as during weight recovery, and both FAT and FFM recoveries reach completion at the same time, i.e., they are synchronized so as to come back exactly to their initial values at the same time, and there is no fat overshooting. By contrast, if *γ* > 1 (i.e., *P*_RF_ < *P*_SS_), then the quantity $(\gamma - 1) \times \Delta W_{{\mathrm{SS}}}$ is positive, resulting in a temporal desynchronization in complete fat and FFM recoveries, with fat recovery reaching completion before that of FFM recovery, with consequential collateral fattening and fat overshooting. A situation when *γ* < 1 is in principle possible (e.g., through special diets, very high-intensity exercise during refeeding, or the use of anabolic compounds), but this will not be considered here. As the lean-fat partitioning ratio $\left( {P_{{\mathrm{SS}}}^{\mathrm{m}}} \right)$ during semistarvation, which is conserved during refeeding, is to a great extent determined by the initial fat percentage %*FAT*_0_ of the subject [43, 44], this motivated us to investigate the relationship between *γ* and %*FAT*_0_. To this end, we revisited here the Minnesota Experiment with specific focus on the analysis of data on body composition of the 12 men who completed the entire study (i.e., including the ad libitum refeeding phase) and showed varying degrees of fat overshooting, as elaborated step-by-step in the section below.

### Revisiting fat overshooting in the Minnesota Experiment

#### Step 1. Relationship between lean-fat partitioning during weight loss and initial adiposity

Before discussing fat overshooting, it is convenient to analyze in more detail the semistarvation mass partition ratio $P_{{\mathrm{SS}}}^{\mathrm{m}}$ as a function of initial adiposity by combining the two following theoretical constraints:

(i) $P_{{\mathrm{SS}}}^{\mathrm{m}} = 1$ if %*FAT*_0_ = 0, since in this situation all mass (virtually all body energy) must be taken from the FFM compartment when the subject loses weight, and
(ii) $P_{{\mathrm{SS}}}^{\mathrm{m}} = 0$ if %*FAT*_0_ = 100, since in this case no mass (virtually no energy) can be taken from the FFM compartment.

With an exponential decreasing function of %*FAT*_0_, one obtains the following expression:

7 $$P_{{\mathrm{SS}}}^{\mathrm{m}} = \frac{{\left( {100 - \% FAT_0} \right)}}{{100}} \times e^{ - c\;\% FAT_0}.$$

**Fig. 2:**
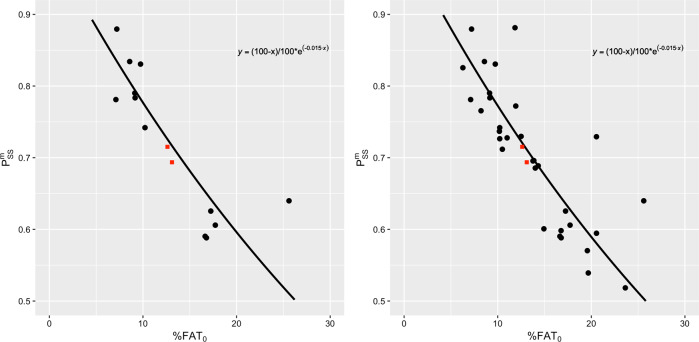
Relationship between the semistarvation mass partition ratio $\left( {P_{{\mathrm{SS}}}^{\mathrm{m}}} \right)$ and the initial percentage body fat (%*FAT*_0_). The expression $P_{{\mathrm{SS}}}^{\mathrm{m}} = (100 - \% FAT_0)/100 \times e^{ - c\;\% FAT_0}$ given in equation 7 is used to fit the data. Left: Including only the 12 subjects that completed the whole Minnesota Experiment. Right: All the 32 subjects that participated to the Minnesota Experiment. The fitted constant is *c* = 0.015. The two red squares correspond to the US Army Ranger data points presented in the section on ‘Applications of the Model’.

In Fig. 2, the fitted constant *c* is given by *c* = 0.015. It is shown that the 12 subjects (out of the 32) who completed all phases of the Minnesota Experiment (i.e., till the end of ad libitum refeeding) are a good representative subset of the 32 subjects that participated in the semistarvation (weight loss) phase and followed by restricted refeeding phase of the experiment, since the fitted curves are almost identical.

#### Step 2. Temporal desynchronization in completion of fat and FFM recoveries

**Fig. 3:**
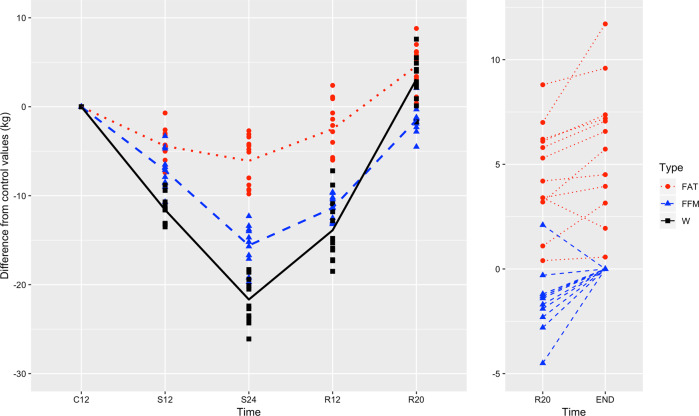
Changes in fat and FFM relative to the control (prestarvation) period (C12), at the end of the 24 weeks of semistarvation (S24) and at the end of refeeding phase (R20) in the 12 men who completed all phases of the Minnesota Experiment. All values are expressed as a difference from the corresponding values during the control (prestarvation) period. More precisely, the values are defined as $FAT_{{\mathrm{time}}} - FAT_{{\mathrm{C12}}}$ for FAT (red circle, dotted line), $FFM_{{\mathrm{time}}} - FFM_{{\mathrm{C12}}}$ for FFM (blue triangle, dashed line) and $W_{{\mathrm{time}}} - W_{{\mathrm{C12}}}$ for W (black square, solid line), for the 12 subjects that completed the Minnesota Experiment. As we shall see later, the final time points END are defined as the times at which the subjects would have completely recovered their initial FFM and are grouped here together for convenience. The lines are added to guide the eyes.

For these 12 men of the Minnesota Experiment who completed the study, the relative changes in fat and FFM (in kg) relative to the control (pre-starvation) period are shown during semistarvation (S12, S24) and refeeding (R12, R20) in Fig. 3 (left panel). The values shown are defined as $FAT_{{\mathrm{time}}} - FAT_{{\mathrm{C12}}}$, $FFM_{{\mathrm{time}}} - FFM_{{\mathrm{C12}}}$ and $W_{{\mathrm{time}}} - W_{{\mathrm{C12}}}$ for FAT, FFM and W, respectively. The data for FAT and FFM are corrected for changes in hydration and relative bone mass, using the correction factor (k) determined in the Minnesota Experiment [35], are as follows: $FAT_{{\mathrm{corrected}}} = k \times FAT_{{\mathrm{raw}}}$, where $k = 0.98,0.91,0.93,0.98$ at time C12, S24, R12 and R20, respectively, and $FFM_{{\mathrm{corrected}}} = k \times FFM_{{\mathrm{raw}}}$, where $k = 0.88,0.92,0.99$ at time S24, R12 and R20, respectively; the raw and corrected data on body composition can be found as Supplementary Tables S1–S3. It is noticed that at R20, while all subjects have fully recovered or overshot their baseline (pre-starvation) body fat levels, with one exception they have not completed their FFM recovery. The inter-individual variability for fat overshoot (range 0–9 kg) and deficit in FFM (−5 to 0 kg) are shown at time R20 in the right-hand panel of Fig. 3. The time-point, which is referred here as ‘END’, corresponds to an extrapolation of each subject’s FFM at R20 to values corresponding to complete (100%) recovery of their FFM; this calculation being made using the linear method for this extrapolation described in a section below.

#### Step 3. Methods in estimating complete FFM recovery and accompanying fat overshoot

In the analysis of fat overshooting, the following holds true:$\Delta W_{{\mathrm{SS}}} = W_{{\mathrm{C12}}} - W_{{\mathrm{S24}}}$ and $\Delta W_{{\mathrm{RF}}} = W_{{\mathrm{END}}} - W_{{\mathrm{S24}}}$. By definition, $FFM_{{\mathrm{END}}} = FFM_{{\mathrm{C12}}}$. To compute the value *FAT*_END_ at the final time END at which the subjects would have completely recovered their initial FFM, one may proceed as follows:

(I) *Null method*: setting $FAT_{{\mathrm{END}}} = FAT_{{\mathrm{R20}}}$ although FFM is not completely recovered at time R20.
(II) *Linear method*: using a linear extrapolation in two steps: first we compute the time END through the equality
  $$FFM_{END} = FFM_{C12} = FFM_{R20} + Slope_{FFM} \times \left( {END - R20} \right),$$
  where $$Slope_{FFM} = \frac{{FFM_{R20} - FFM_{R12}}}{{R20 - R12}}.$$
  Then we set $FAT_{END} = FAT_{R20} + Slope_{FAT} \times \left( {END - R20} \right),$
  where $$Slope_{FAT} = \frac{{FAT_{R20} - FAT_{R12}}}{{R20 - R12}}$$.
(III) *Individual mass partition ratio method:* using the equation 2 for $P_{{\mathrm{SS}}}^{\mathrm{m}}$ one may write
  8 $$FAT_{{\mathrm{END}}} = FAT_{{\mathrm{R20}}} + \Delta FFM \cdot \left( {1/P_{{\mathrm{SS}}}^{\mathrm{m}} - 1} \right),$$
  where $\Delta FFM = FFM_{{\mathrm{C12}}} - FFM_{{\mathrm{R20}}}$ and each individual $P_{{\mathrm{SS}}}^{\mathrm{m}}$ is computed during the semistarvation period.
(IV) *Fitted mass partition ratio method*: using equation 8 in the method III above but with the mass partition ratio $\left( {P_{{\mathrm{SS}}}^{\mathrm{m}}} \right)$ obtained from the exponential fit given in equation 7, and shown in Fig. 2.

**Fig. 4:**
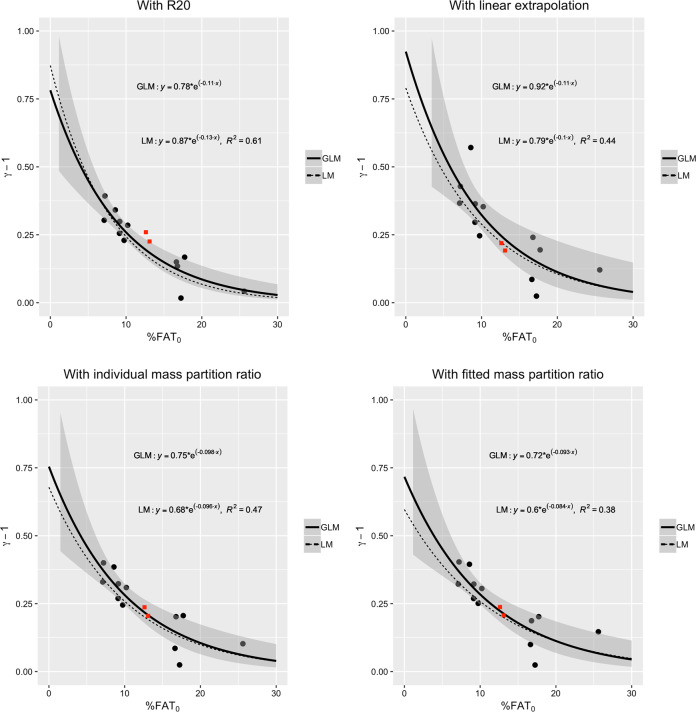
The values for (*γ* − 1) are plotted vs. the initial fat percentage %*FAT*_0_. Two exponential fits are shown: a generalized linear model (GLM) with 95% confidence intervals (solid line), and a linear model (LM), with *R*^2^ values (dotted line). The four figures correspond to the four methods presented in the main text for computing the value *FAT*_END_ at the final time END at which the subjects would have completely recovered their initial FFM. The two red squares correspond to the US Army Ranger data points presented in the section on ‘Applications of the Model’.

As shown in Fig. 4, the parameter *γ* is well associated with the initial fat percentage %*FAT*_0_. Indeed, the data points clearly show that (*γ* − 1) decreases with %*FAT*_0_. In subjects going through a weight cycle under “ordinary” conditions, we expect the FAT overshoot to be positive for all values of %*FAT*_0_. Recalling equation 6, we thus expect that *γ* is greater than 1, or equivalently that (*γ* − 1) is greater than 0 for all values of %*FAT*_0_. These considerations motivate us to model (*γ* − 1) as an exponential function of %*FAT*_0_:

9 $$\gamma - 1 = a\,e^{ - b\,{\mathrm{\% }}FAT_0}$$

where the constants *a* and *b* are determined by fitting this model to the data.

Their exact values depend on the method used for computing the values *FAT*_END_ when the FFM has been completely recovered and on the type of statistical regression used. As explained previously, we consider four methods for computing *FAT*_END_. In Fig. 4, we show the fits obtained from a generalized linear model (GLM) with 95% confidence intervals (solid line) and from a linear model (LM), with *R*^2^ values (dotted line). Following the performance of diagnostics tests to analyze the residuals, it is found that the GLM satisfies the major assumptions of regression analysis better than the LM, especially concerning the normality assumption of the error terms. Nevertheless, as shown in Fig. 4, both models give curves that are close to each other. In simple terms, the main difference between the above LM and GLM approaches to estimate the best parameter values for the constants *a* and *b* is the way the error term is handled. In the LM method, we write *y* = *a* *×* *e*^*bx*^ *×* *ε*, where ε is a random error variable that follows a log-normal distribution with parameters μ = 0 and *σ*^2^. Taking the logarithm on both sides of the equality gives $\log \left( y \right) = \log \left( a \right) + b\,x + {\mathrm{log}}(\varepsilon)$, which corresponds to the linear model $y^\prime = \beta _0 + \beta _1\,x^\prime + \varepsilon ^\prime$, where $y^\prime = {\mathrm{log}}(y)$, *x*′ = *x*, $\beta _0 = {\mathrm{log}}(a)$, *β*_1_ = b and $\varepsilon ^\prime = {\mathrm{log}}(\varepsilon )$ is a random error variable that follows a normal distribution with mean μ = 0 and variance *σ*^2^. Note that in the LM method, the error term *ε* is multiplied with the exponential function *y* = *a* *× e*^*bx*^. In the GLM method, the error term is instead added to the exponential function. Formally, we write *y* = *a* *×e*^*bx*^ + *ε*, where ε is a random error variable that follows a normal distribution with mean 0 and variance *σ*^2^. As a consequence, the GLM admits the possibility of the value *y* = 0 (which is excluded in LM) and assumes that the variance *V*(*y*) is constant, while the LM method assumes that *V*(*y*) varies with *x*. Finally, note that the LM method admits exact analytical solutions for the model parameters *a* and *b*, while the GLM method requires numerical optimization algorithms to find the best values (the maximum likelihood estimates) for *a* and *b*.

#### Step 4. Relationship between *γ* and %*FAT*_0_

From equation 9, we have the approximation $\gamma - 1 = a\;e^{ - b\;\% FAT_0}$, where the exact values of the fitted constants, *a* and *b*, depend on the method used for computing the values for FAT when FFM has been completely recovered. For example in Fig. 4, we observe that by using the linear extrapolation method and a generalized linear model, one obtains *a* = 0.92 and *b* = 0.11. In other words, the factor *γ* may be well approximated with the following expression:

10 $$\gamma = 1 + a\;e^{ - b\;\% FAT_0}.$$

### Model prediction of fat overshoot

Combining this expression with equation 6, one may predict the fat overshoot in a subject with initial fat percentage %*FAT*_0_ and having lost weight Δ*W*_SS_ through semistarvation:

11 $$FAT\;Overshoot = a\;e^{ - b\;\% FAT_0} \times \Delta W_{{\mathrm{SS}}}.$$

**Fig. 5:**
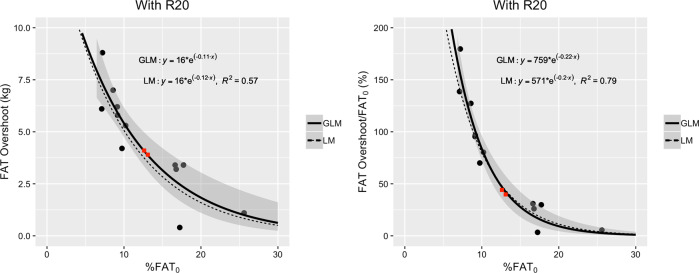
Relationship between fat overshoot and initial adiposity (%*FAT*_0_), with fat overshoot expressed in absolute term (kg fat) in left panel, and expressed as a percentage of baseline body fat (*FAT*_0_) in right panel. The two red squares correspond to the US Army Ranger data points presented in the section on ‘Applications of the Model’.

It is to be noted that in the Minnesota Experiment, the percentage weight loss (25–29%), as well as the absolute amount of weight loss (18.3–26.1 kg) were not too different among the subjects. In this case, with the assumption that ∆Wss is a constant, the relationship between FAT overshoot vs. %*FAT*_0_ can be plotted as shown in Fig. 5, rather than as FAT overshoot/∆Wss vs. %FAT_0_ as we would expect from the model.

### Applications of the model

#### US Army Rangers fat overshooting

As a first application, the model described here is used to make predictions for the fat overshoot in US Army Rangers who regained weight after 8 weeks of energy deficit resulting from intense training in a multistressor environment [49, 50], which will then be compared with actual observed (or measured) overshoot values. The data are presented *as red square symbols in* Figs. 2, 4, 5 and in Supplementary Table S3. We have applied the same correction procedure as for the Minnesota data. In the publication of these studies [49, 50], only the mean values are accessible. The FAT overshoot is obtained by using the four methods presented previously to ensure that $FFM_2 = FFM_0$; the data are provided in Supplementary Table S4. The predicted FAT overshoot is computed with equation 11 using the four methods and the GLM. As shown in Table S4, the predictions are very close to the observed values.

#### Weight cycling from leanness to fatness

**Fig. 6:**
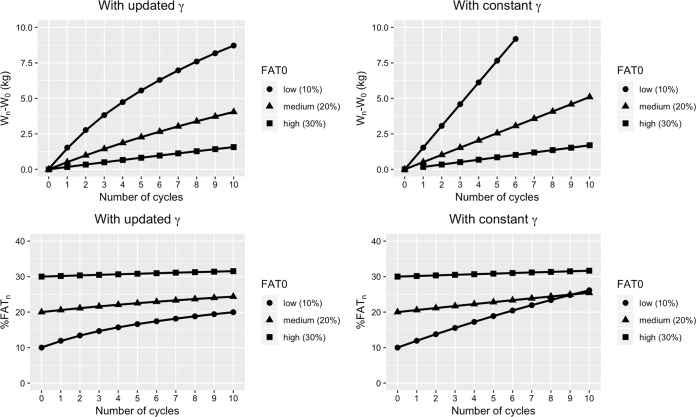
Simulation of gain in body weight and body fat percentage in response to 10 weight cycles. Subjects of weight 70 (kg) and initial fat percentage 10% (low) or 20% (medium) or 30% (high) lose 5 (kg) at each cycle. We use the equations 6 and 10 and show the weight difference $W_n - W_0 = FAT_n - FAT_0$, where *n* = 0, …., 10 is the number of cycles completed by the subjects. The *γ* values are obtained by using the linear method for computing *FAT*_END_ and the GLM for the fit (*a* = 0.92, *b* = 0.11). Top left: the values of *γ* are updated at each cycle, according to the new fat percentage, leading to a decrease in fat overshoot at each new cycle. Top right: the values of *γ* are all equal to the initial value of *γ* corresponding to the initial fat percentage, leading to a constant fat overshoot at each cycle and a linear increase in weight. Bottom panels: the fat percentage of the subjects at each cycle.

The model is now used to predict body fat accumulation in three hypothetical subjects of identical weight (70 kg) but with different initial body fat%: 10% (low), 20% (medium) and 30% (high) who go through several successive weight cycles, with the lost and regain of 5 kg over each cycle (Fig. 6). To determine their weights at the end of the first cycle, we will use the equation 6 with the relation 10 for *γ*. To compute their weight at the end of the next cycles, we need to know how the values for *γ* are updated at the end of each cycle. In other words, after the fat overshoot in one cycle, is the intrinsic lean-fat partitioning over the next cycle the same as in the previous cycle or is it diminished as adiposity has increased, i.e., it moves to the right of the inverse exponential relating the lean-fat partitioning to the initial adiposity [41, 42, 44]. This is unknown, and as it may depend on the timespan between two successive cycles, we shall consider two simple possibilities:

(i) The values for *γ* are updated at each cycle according to the new body fat percentage and
(ii) The values for *γ* are kept constant (the initial value of *γ* corresponding to the initial fat percentage) at all cycles. For simplicity, the *γ* values are obtained here by using the linear method for computing *FAT*_END_ and the GLM for the fit (*a* = 0.92, *b* = 0.11).

In the top panels of Fig. 6, we show the weight difference *W*_*n*_ − *W*_0_ for 10 weight cycles. Note that since the FFM values are the same at the beginning and at the end of each cycle $\left( {FFM_n = FFM_0} \right)$, we actually have $W_n - W_0 = FAT_n - FAT_0$. On the left-hand side, we suppose that the values for *γ* are updated at each cycle, according to the new body fat%, leading to a decrease in fat overshoot at each new cycle. On the right-hand side, we assume that the values of *γ* are all equal to the initial value of *γ* corresponding to the initial fat%, leading to a constant fat overshoot at each cycle and a linear increase in weight. In the bottom panels of Fig. 6, we show the body fat% of the subjects at each cycle. It is shown that the subject with the low initial fat% (10%) gets closer to the one with medium initial fat% (20%) as the number of cycles increases, and may even overtake him in the constant *γ* situation. It can also be observed that the subject with high initial fat% (30%) remains essentially at the same fat% during the 10 weight cycles; the predictions by our model of little or no fat overshooting in dieters with obesity is in accord with studies in which individuals with obesity when subjected to one cycle [51] or three successive cycles [52] of dieting failed to show altered body composition. More recent support for the notion that the parameter *γ* is ~1 for people with very high initial body fat can also be derived from the “Biggest Loser” study in which the subjects lost and regained body fat and FFM in the same proportion [53]. By contrast, even if the values for *γ* are updated at each cycle in a lean dieter subjected to multiple weight cycles (with the amount of fat overshoot decreasing with each successive cycle), the cumulative amount of fat overshoot over several cycles will nonetheless result in the deposition of a substantial amount of excess of body fat.

In addition to uncertainties for updating of the values for *γ* at each cycle, diet composition may also be a factor that can influence the asynchronous recovery of body fat and FFM and hence the factor *γ*. Indeed, an increased dietary fat intake was reported during weight recovery relative to baseline in the Army Ranger studies [49] and during the ‘ad libitum refeeding’ phase of the Minnesota Experiment [35]. Using a mathematical model of macronutrient balance, Hall [54] showed that the asynchronous recovery of body fat vs. FFM in the Minnesota Experiment may have been due to such changes in diet composition; furthermore, this model predicted recovery of the original body composition in the Minnesota men upon returning to the pre-starvation diet and physical activity, albeit after an extended duration (>1 year) of consuming the baseline diet [54]. It should be noted, however, that excess dietary fat intake is unlikely to be the sole explanation for the asynchronous recovery of body fat vs. FFM in the Minnesota Experiment. Indeed, in the earlier phase of controlled refeeding lasting 12 weeks when dietary fat intake (in both absolute and relative terms) was actually lower than during the baseline period, a disproportionately faster recovery of body fat relative to FFM was also observed [35]; this was attributed to a sustained reduction of thermogenesis contributing to accelerated fat storage [43, 45]. Overall, in our model presented here, it should be underlined that the parameter *γ* may not only depend upon the time factor and the period of time between cycling pattern but also upon dietary composition.

## Concluding remarks

While the prevalence of dieting to lose weight is more common in those people with obesity or overweight, it is substantial (and rising) in normal-weight population groups that include females and males, young and older adults, children and adolescents who perceive themselves as being too fat, as well as among athletes in weight-sensitive sports and among those in occupations where a slim image is professionally an advantage [34]. In these persons with initially normal weight, dieting attempts may predispose one to or represent another predisposition to future weight gain. Indeed, the loss of body weight has been shown to induce both metabolic and behavioral changes by which the body struggles to regain the weight [55, 56]. In the context of dieting to lose weight, personal attitudes toward dieting, social pressure to diet or body image, as well as post-slimming preoccupation with food, disinhibition and moral self-licensing for obesity-prone behavior may also act as a driver for weight regain, and contribute to fat overshooting. Obviously this mathematical model cannot account for all the determinants involved in fat overshooting, but represents merely the impact of an autoregulatory component and it has the merit to be simple and useful in practice, since it depends only on the measurable parameters %*FAT*_0_ and Δ*W*_SS_.

## Supplementary information

Supplementary Table S1

Supplementary Table S2

Supplementary Table S3

Supplementary Table S4
