# Supplementary material for: How dieting might make some fatter: modeling weight cycling toward obesity from a perspective of body composition autoregulation
Source: Int J Obes (Lond). 2020 Feb 25;44(6):1243–53. doi: 10.1038/s41366-020-0547-1 (PMC7260129; doi:10.1038/s41366-020-0547-1)
Supplement: Supplementary file 1 — Supplementary Table S1 [file 41366_2020_547_MOESM1_ESM.pdf]

Supplementary **Table S1:** Body fat (FAT) in kg and Fat-free mass (FFM) in kg in the Minnesota Experiment

C12: at of Control (baseline) period
S12: at week 12 of semistarvation
S24: at week 24 of semistarvation
R12: at week 12 of refeeding
R20: at week 20 of refeeding
NA : data not available

Subject no.	FAT_C12	FAT_S12	FAT_S24	FAT_R12	FAT_R20	FFM_C12	FFM_S12	FFM_S24	FFM_R12	FFM_R20
122	4.2	0	0	0	*NA*	61.2	52.1	47.4	51.6	*NA*
123	6.7	2.3	2.0	2.8	12.1	58.0	53.5	50.1	51.4	56.8
119	11.2	6.2	1.9	5.5	14.7	55.1	48.3	47.2	47.7	57.3
120	14.1	8.8	3.4	5.1	*NA*	56.7	48.9	48.2	48.6	*NA*
129	6.1	1.3	2.0	4.9	12	59.5	54.8	50.2	52.4	57.9
130	13.8	8.8	9.4	7.5	*NA*	52.2	48.7	44.2	48.4	*NA*
126	17.5	8.4	5.6	6.1	*NA*	66.1	60.7	55.0	58.3	*NA*
127	5.6	2.9	2.3	3.7	12.8	58.6	49.6	47	49.4	54.2
22	9.5	3.5	3.0	6.1	*NA*	55.6	50.3	46.4	47.9	*NA*
23	12.1	5.2	3.4	6.4	12.5	57.1	50.6	48.0	49.8	56.9
19	9.0	2.7	2.0	4.2	*NA*	61.6	54.8	48.4	51.3	*NA*
20	6.7	3.6	0.6	4.1	*NA*	58	50.3	47.4	50.0	*NA*
29	8.6	5.1	6.2	6.7	*NA*	62.5	49.4	47.3	52.1	*NA*
30	8.3	6.5	3.5	7.6	*NA*	59.8	51.1	48.9	50.8	*NA*
26	6.6	0.5	1.5	4.0	13	64.7	57.6	51.6	56.2	63.6
27	11.4	2.5	1.0	3.8	*NA*	63.8	58.3	54.7	54.8	*NA*
4	4.5	1.4	0.0	5.7	10.7	57.5	49.0	47.4	52.0	55.7
5	19.4	9.5	5.1	8.1	*NA*	61.4	55.1	52.0	54.6	*NA*
1	10.8	3.6	2.7	5.5	*NA*	66.4	61.3	54.3	58.5	*NA*
2	12.5	7.7	2.7	7.1	15.8	61	52.4	53.2	55.1	58.8
11	11.3	6.1	2.3	6.6	*NA*	55.1	48.3	47.3	51.1	*NA*
12	11.4	4.8	4.0	5.8	*NA*	69.6	63.9	59.2	61.6	*NA*
8	5.4	0	0	4.4	*NA*	59.2	53.9	47.5	53.5	*NA*
9	8.2	0.6	2.4	5.5	*NA*	64.7	59.8	55.7	58.7	*NA*
104	5.0	4.2	2.4	6.4	14	63.1	52.4	49.2	54.7	62.0
105	7.3	3.9	0.7	7.9	*NA*	61.4	52.6	51.1	53.1	*NA*
101	11.6	4.0	3.7	11.5	15.1	52.9	49.6	46.0	46.5	51.8
102	13.6	7.4	3.6	11.2	*NA*	54.3	48.9	48.3	51.0	*NA*
111	9.0	2.9	3.0	9.9	*NA*	54	49.2	46.1	50.2	*NA*
112	6.1	2.8	3.2	9.0	10.4	55.6	47.7	45.8	50.1	54.3
108	6.9	3.8	2.0	6.3	*NA*	60	53.6	52.1	53.9	*NA*
109	20.6	15.2	11.9	15.3	21.7	58.7	50.2	47.6	50.3	56.0
