# Supplementary material for: How dieting might make some fatter: modeling weight cycling toward obesity from a perspective of body composition autoregulation
Source: Int J Obes (Lond). 2020 Feb 25;44(6):1243–53. doi: 10.1038/s41366-020-0547-1 (PMC7260129; doi:10.1038/s41366-020-0547-1)
Supplement: Supplementary file 2 — Supplementary Table S2 [file 41366_2020_547_MOESM2_ESM.pdf]

Supplementary **Table S2**: Body fat (FAT) in kg and Fat-free mass (FFM) in kg, after corrections for excess hydration and relative bone mass
C12: at of Control (baseline) period
S12: at week 12 of semistarvation
S24: at week 24 of semistarvation
R12: at week 12 of refeeding
R20: at week 20 of refeeding
NA : data not available

Subject no.	FAT_C12	FAT_S12	FAT_S24	FAT_R12	FAT_R20	FFM_C12	FFM_S12	FFM_S24	FFM_R12	FFM_R20
122	4.1	0	0	0	*NA*	61.2	52.1	41.8	47.4	*NA*
123	6.6	2.3	1.8	2.6	11.9	58.0	53.5	44.2	47.2	56.7
119	11.0	6.2	1.7	5.1	14.4	55.1	48.3	41.7	43.8	57.2
120	13.8	8.8	3.1	4.7	*NA*	56.7	48.9	42.5	44.6	*NA*
129	6.0	1.3	1.8	4.6	11.8	59.5	54.8	44.3	48.1	57.8
130	13.5	8.8	8.6	7.0	*NA*	52.2	48.7	39.0	44.5	*NA*
126	17.1	8.4	5.1	5.7	*NA*	66.1	60.7	48.5	53.6	*NA*
127	5.5	2.9	2.1	3.4	12.5	58.6	49.6	41.5	45.4	54.1
22	9.3	3.5	2.7	5.7	*NA*	55.6	50.3	41.0	44.0	*NA*
23	11.9	5.2	3.1	6.0	12.3	57.1	50.6	42.4	45.7	56.8
19	8.8	2.7	1.8	3.9	*NA*	61.6	54.8	42.7	47.1	*NA*
20	6.6	3.6	0.5	3.8	*NA*	58.0	50.3	41.8	45.9	*NA*
29	8.4	5.1	5.6	6.2	*NA*	62.5	49.4	41.7	47.9	*NA*
30	8.1	6.5	3.2	7.1	*NA*	59.8	51.1	43.2	46.7	*NA*
26	6.5	0.5	1.4	3.7	12.7	64.7	57.6	45.5	51.6	63.4
27	11.2	2.5	0.9	3.5	*NA*	63.8	58.3	48.3	50.3	*NA*
4	4.4	1.4	0	5.3	10.5	57.5	49.0	41.8	47.8	55.6
5	19.0	9.5	4.6	7.5	*NA*	61.4	55.1	45.9	50.2	*NA*
1	10.6	3.6	2.5	5.1	*NA*	66.4	61.3	47.9	53.7	*NA*
2	12.3	7.7	2.5	6.6	15.5	61.0	52.4	47.0	50.6	58.7
11	11.1	6.1	2.1	6.1	*NA*	55.1	48.3	41.7	46.9	*NA*
12	11.2	4.8	3.6	5.4	*NA*	69.6	63.9	52.2	56.6	*NA*
8	5.3	0	0	4.1	*NA*	59.2	53.9	41.9	49.1	*NA*
9	8.0	0.6	2.2	5.1	NA	64.7	59.8	49.2	53.9	*NA*
104	4.9	4.2	2.2	6.0	13.7	63.1	52.4	43.4	50.2	61.9
105	7.2	3.9	0.6	7.3	*NA*	61.4	52.6	45.1	48.8	*NA*
101	11.4	4.0	3.4	10.7	14.8	52.9	49.6	40.6	42.7	51.7
102	13.3	7.4	3.3	10.4	*NA*	54.3	48.9	42.6	46.8	*NA*
111	8.8	2.9	2.7	9.2	*NA*	54.0	49.2	40.7	46.1	*NA*
112	6.0	2.8	2.9	8.4	10.2	55.6	47.7	40.4	46.0	54.2
108	6.8	3.8	1.8	5.9	*NA*	60.0	53.6	46.0	49.5	*NA*
109	20.2	15.2	10.8	14.2	21.3	58.7	50.2	42.0	46.2	55.9
