# Supplementary material for: How dieting might make some fatter: modeling weight cycling toward obesity from a perspective of body composition autoregulation
Source: Int J Obes (Lond). 2020 Feb 25;44(6):1243–53. doi: 10.1038/s41366-020-0547-1 (PMC7260129; doi:10.1038/s41366-020-0547-1)
Supplement: Supplementary file 3 — Supplementary Table S3 [file 41366_2020_547_MOESM3_ESM.pdf]

Supplementary **Table S3:** US Army Rangers - Body composition data

Raw data for the US Army Rangers

	FAT0	FAT1	FAT2	FFM0	FFM1	FFM2
Nindl et al. (1997) ref. 43	10.0	5.0	14.0	65.0	60.0	66.0
Friedl et al. (2000) ref. 44	9.5	5.3	13.7	64.4	60.2	65.5

Corrected data for the US Army Rangers (*corrected for excess hydration & relative bone mass*)

ID	FAT0	FAT1	FAT2	FFM0	FFM1	FFM2
Nindl et al. (1997) ref. 43	9.8	4.5	13.7	65.0	53.0	65.8
Friedl et al. (2000) ref. 44	9.3	4.8	13.4	64.4	53.1	65.3
