# Supplementary material for: How dieting might make some fatter: modeling weight cycling toward obesity from a perspective of body composition autoregulation
Source: Int J Obes (Lond). 2020 Feb 25;44(6):1243–53. doi: 10.1038/s41366-020-0547-1 (PMC7260129; doi:10.1038/s41366-020-0547-1)
Supplement: Supplementary file 4 — Supplementary Table S4 [file 41366_2020_547_MOESM4_ESM.pdf]

Supplementary **Table S4:** The US Army Rangers FAT overshoot observations and . predictions using equation [8] with the four methods (I, II, III, IV) and the GLM

a) *Using Data Corrected for excess hydration and relative bone mass*

Ref	%FAT	*Δ Wss* (kg)	Prediction method	FAT overshoot (kg)	Predicted FAT overshoot (kg)
43	13.1	17.3	I	3.9	3.2 [2.5, 3.9]
			II	3.3	3.8 [2.6, 5.4]
			III	3.5	3.6 [2.7, 4.5]
			IV	3.6	3.7 [2.8, 4.6]
44	12.6	15.8	I	4.1	3.1 [2.4, 3.7]
			II	3.5	3.6 [2.7, 5.1]
			III	3.7	3.4 [2.7, 4.2]
			IV	3.8	3.5 [2.7, 4.3]

b) *Using raw data*

Ref.	%FAT	*Δ Wss* (kg)	Prediction method	FAT overshoot (kg)	Predicted FAT overshoot (kg)
43	13.3	10	I	4.0	1.8
			II	2.5	2.1
			III	3.0	2.0
			IV	3.6	2.1
44	12.9	8.4	I	4.2	1.6
			II	2.5	1.9
			III	3.1	1.8
			IV	3.8	1.8
